# Supplementary material for: Parameter Estimation and Identifiability in Kinetic Flux Profiling Models of Metabolism
Source: Bull Math Biol. 2024 Nov 27;87(1):7. doi: 10.1007/s11538-024-01386-x (PMC11602815; doi:10.1007/s11538-024-01386-x)
Supplement: Supplementary file 1 — (pdf 5816 KB) [file 11538_2024_1386_MOESM1_ESM.pdf]

1 Steady-State Problem - Supplementary Information

1.1 Irreversible Two-Metabolite Similar Turnover Rates Example

1.1.1 Only Steady-State Data

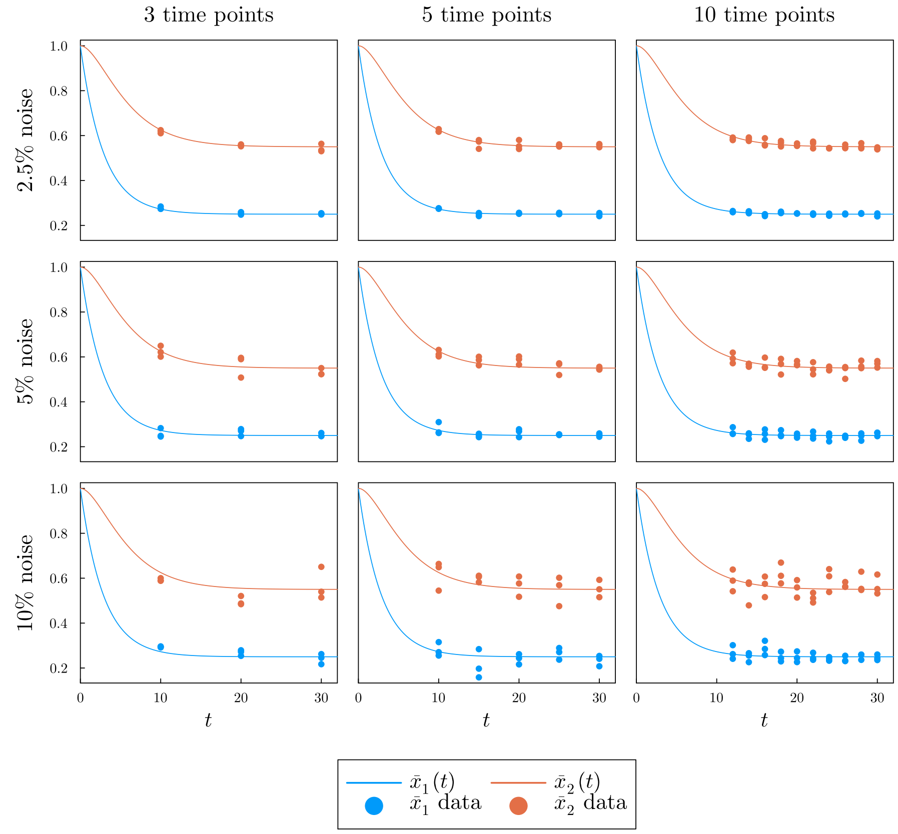

Fig. 1 Solutions and simulated noisy data for the irreversible two-metabolite model with similar turnover rates when data is collected only at steady state of the system. The simulated data are taken from solutions of the system with parameters $k_1 = 7/20$, $\alpha_1 = 1/4$, $k_2 = 3/10$, $\alpha_2 = 2/5$ at 3, 5, or 10 equally distributed time points. At each time point, three noisy data points are simulated by adding random normally distributed noise to the true solutions with a standard deviation of 2.5, 5, or 10% of the true value

In fig. 1, we see that the experimental measurements are only taken while $\bar{x}_1$ and $\bar{x}_2$ approach their steady-state values. Since the experimental data captures limited

information about the system we expect to get poor estimates for the turnover rates. On the other hand, sufficient information about the steady states are provided and we expect to accurately recover the proportional parameters α_1 and α_2 regardless of the number of time points and level of noise.

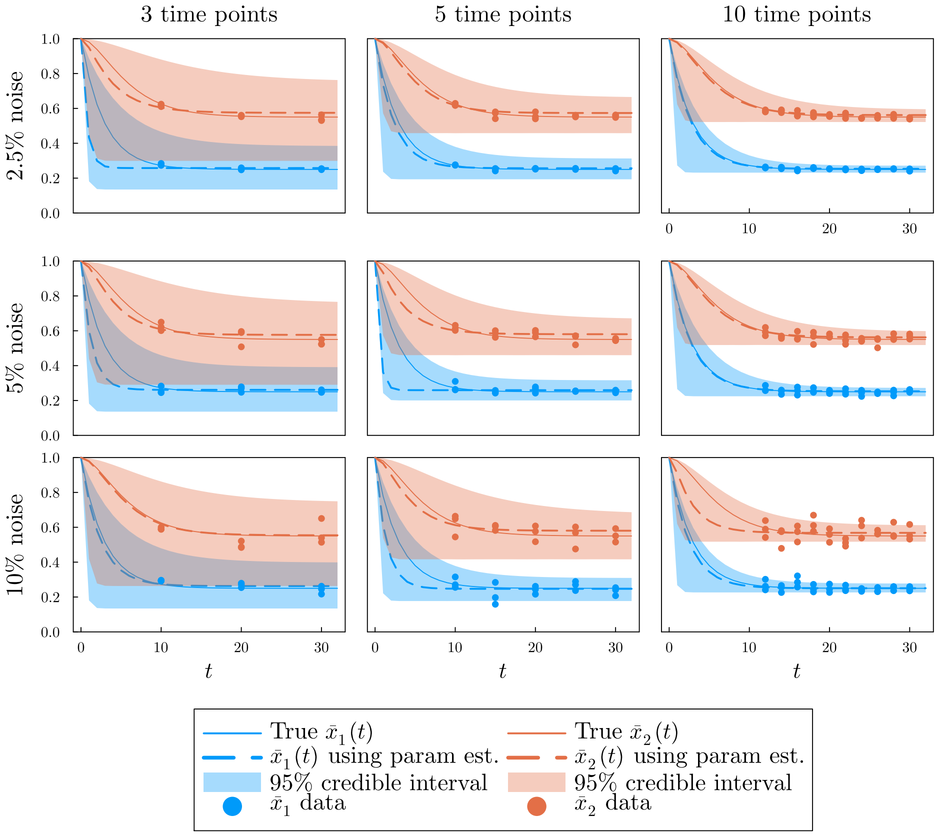

Fig. 2 Comparing solutions using the values produced from Bayesian parameter estimation to the experimental data and true solutions for the irreversible two-metabolite model with similar turnover rates when data is collected only at the system's steady state. The simulated data are taken from solutions of the system with parameters $k_1 = 7/20$, $\alpha_1 = 1/4$, $k_2 = 3/10$, $\alpha_2 = 2/5$ at 3, 5, or 10 equally distributed time points. At each time point, three noisy data points are simulated by adding random normally distributed noise to the true solutions with a standard deviation of 2.5, 5, or 10% of the true value. The 95% credible interval was created by plotting the region between the solution with the 2.5% and the 97.5% quartiles of the posterior distributions of the parameters

In fig. 2, we see that the solution using the parameter estimates does not fit the true solution during the decay of $\bar{x}_1$ and $\bar{x}_2$. The 95% credible interval is the region where 95% trajectories produced by the samples from the posterior parameter distributions would be. Also, the 95% credible interval tightens around the true solution at steady state as we increase the number of time points and decrease the level of noise in the measurements.

1.1.2 Decay and Steady-State Data

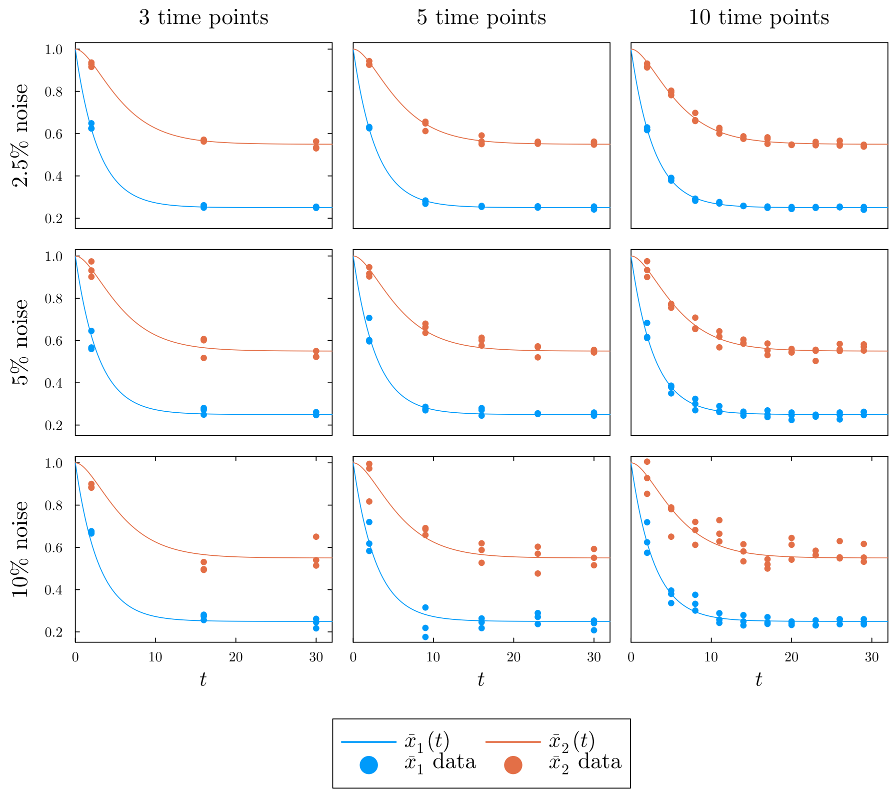

Fig. 3 Solutions and simulated noisy data for the irreversible two-metabolite model with similar turnover rates. The simulated data are taken from solutions of the system with parameters $k_1 = 7/20$, $\alpha_1 = 1/4$, $k_2 = 3/10$, $\alpha_2 = 2/5$ at 3, 5, or 10 equally distributed time points. At each time point, three noisy data points are simulated by adding random normally distributed noise to the true solutions with a standard deviation of 2.5, 5, or 10% of the true value

In fig. 3, we see that the experimental measurements are taken while both $\bar{x}_1$ and $\bar{x}_2$ are still decaying and as both approach their steady-state values. Since the experimental data captures information about the decay rates and steady-state values we expect to get accurate estimates of all parameter values with low uncertainty given sufficient time points and little noise.

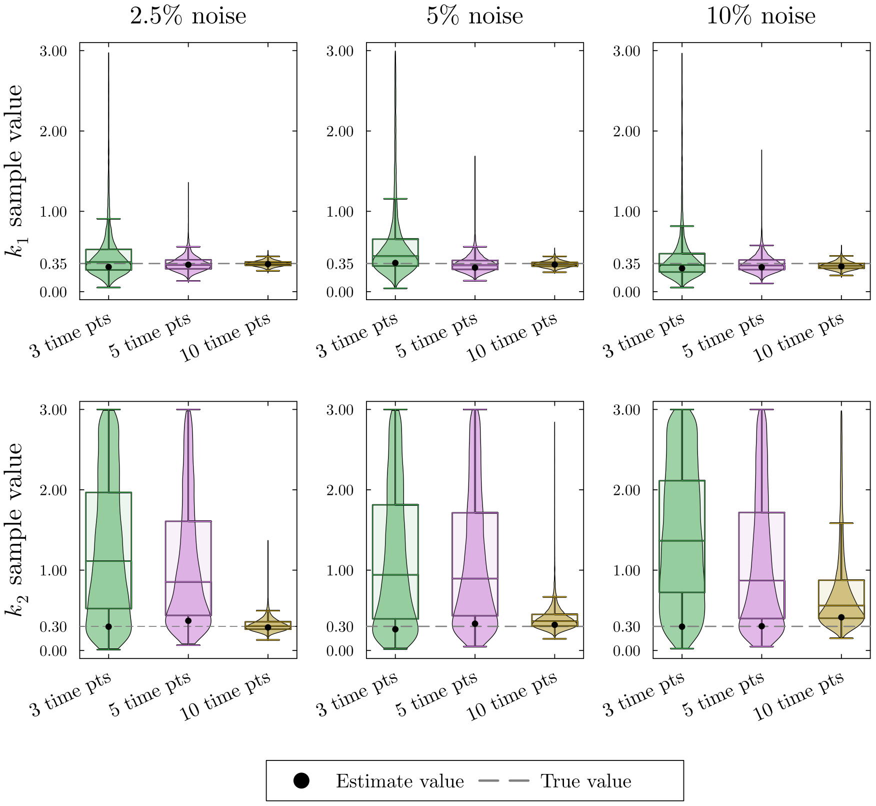

Fig. 4 Violin and box plots for posterior distributions of k_1 and k_2 for the irreversible two-metabolite model with similar turnover rates containing both decay and steady-state data. The simulated data are taken from solutions of the system with parameters $k_1 = 7/20$, $\alpha_1 = 1/4$, $k_2 = 3/10$, $\alpha_2 = 2/5$ at 3, 5, or 10 equally distributed time points. At each time point, three noisy data points are simulated by adding random normally distributed noise to the true solutions with a standard deviation of 2.5, 5, or 10% of the true value. Since k_1, and k_2 are turnover rates, we use the naive prior distributions $k_1 \sim U(0, 3)$, $k_2 \sim U(0, 3)$. The mode of the posterior samples is shown as a dot and is taken as the estimated value. Outliers are excluded in the box plots

From fig. 4, we see that the Bayesian estimation method condensed the sample values near the true parameter values for both parameters. Regardless of the noise level, the parameter estimation increases in accuracy with the inclusion of more time points. The estimated value of the posterior distributions approaches the true parameter value as the number of time points increases. We also note that the uncertainty in the estimation decreases with the increase in time points. With 10 time points and 2.5% noise, we receive the best estimates with the least uncertainty for both k_1 and k_2.

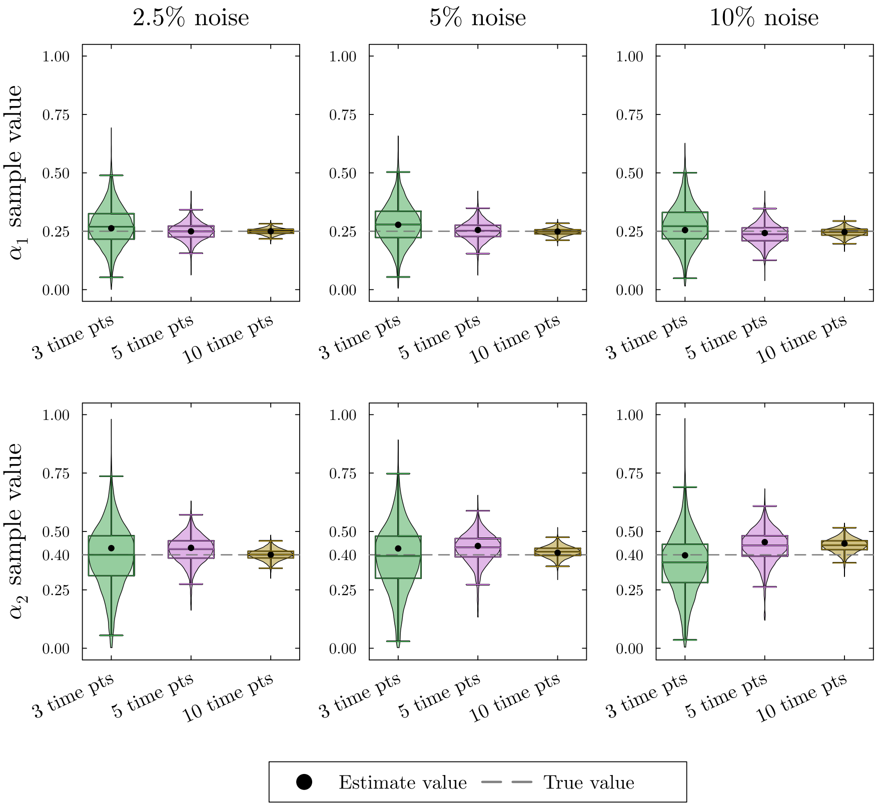

Fig. 5 Violin and box plots for posterior distributions of α_1 and α_2 for the irreversible two-metabolite model with similar turnover rates. The simulated data are taken from solutions of the irreversible two-metabolite system with parameters $k_1 = 7/20$, $\alpha_1 = 1/4$, $k_2 = 3/10$, $\alpha_2 = 2/5$ at 3, 5, or 10 equally spaced time points. At each time point, three noisy data points are simulated by adding random normally distributed noise to the true solutions with a standard deviation of 2.5, 5, or 10% of the true value. Since α_1, and α_2 are proportions, we use the natural naive prior distributions $\alpha_1 \sim U(0, 1)$, $\alpha_2 \sim U(0, 1)$. The mode of the posterior samples is shown as a dot and is taken as the estimated value. Outliers are excluded in the box plots

In the violin plots in fig. 5, we see the method consistently provides accurate estimates of both α_1 and α_2 as the mode of the posterior distributions. The spread of the violin plots or box plots is a visual indicator of the uncertainty in our estimate. The comparison across number of time points shows, not surprisingly, that more time points generally provides a more accurate and less variable estimate. The results are relatively insensitive to the level of noise in the data. Increasing the noise level only slightly increases the error and the uncertainty in the estimate.

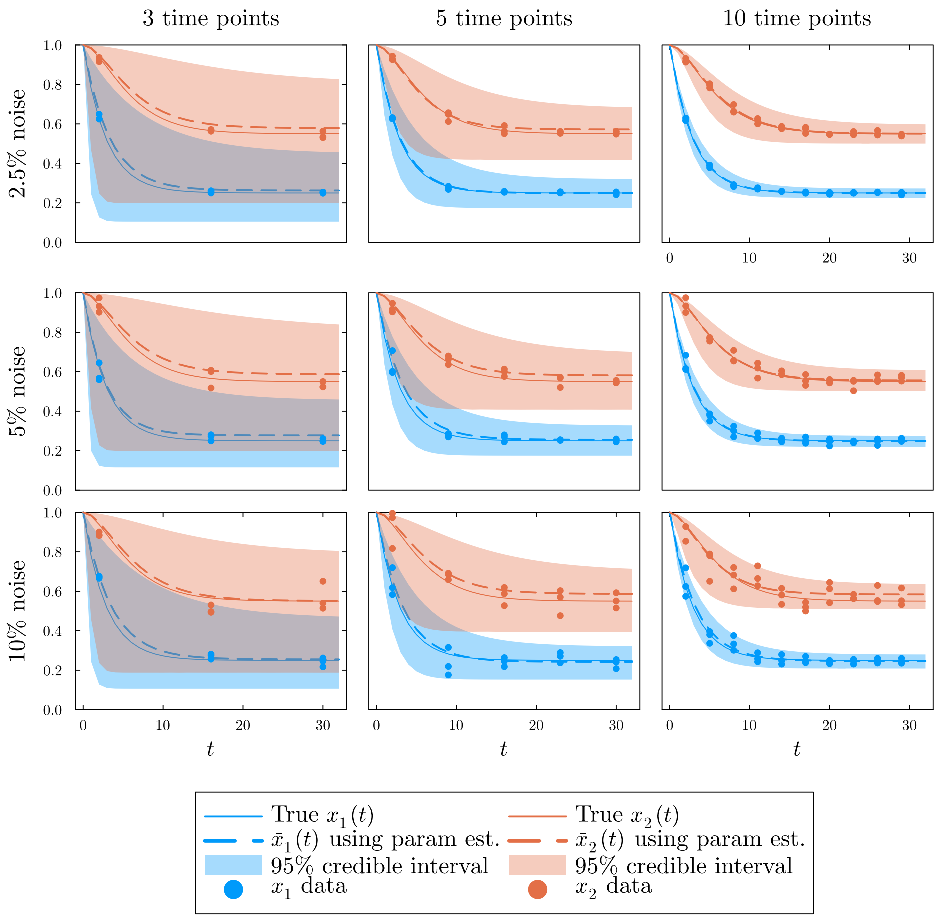

Fig. 6 Comparing solutions using the values produced from Bayesian parameter estimation to the experimental data and true solutions for the irreversible two-metabolite model with similar turnover rates. The simulated data are taken from solutions of the system with parameters $k_1 = 7/20$, $\alpha_1 = 1/4$, $k_2 = 3/10$, $\alpha_2 = 2/5$ at 3, 5, or 10 equally distributed time points. At each time point, three noisy data points are simulated by adding random normally distributed noise to the true solutions with a standard deviation of 2.5, 5, or 10% of the true value. The 95% credible interval was created by plotting the region between the solution with the 2.5% and the 97.5% quartiles of the posterior distributions of the parameters

In fig. 6, we see that the solution using the parameter estimates approaches the true solution as we increase the number of time points. The 95% credible interval is the region where 95% trajectories produced by the samples from the posterior parameter distributions would be. Also, the 95% credible interval tightens around the true solution as we increase the number of time points and decrease the level of noise in the measurements.

1.2 Reversible Two-Metabolite Similar Turnover Rates Example

1.2.1 Only Steady-State Data

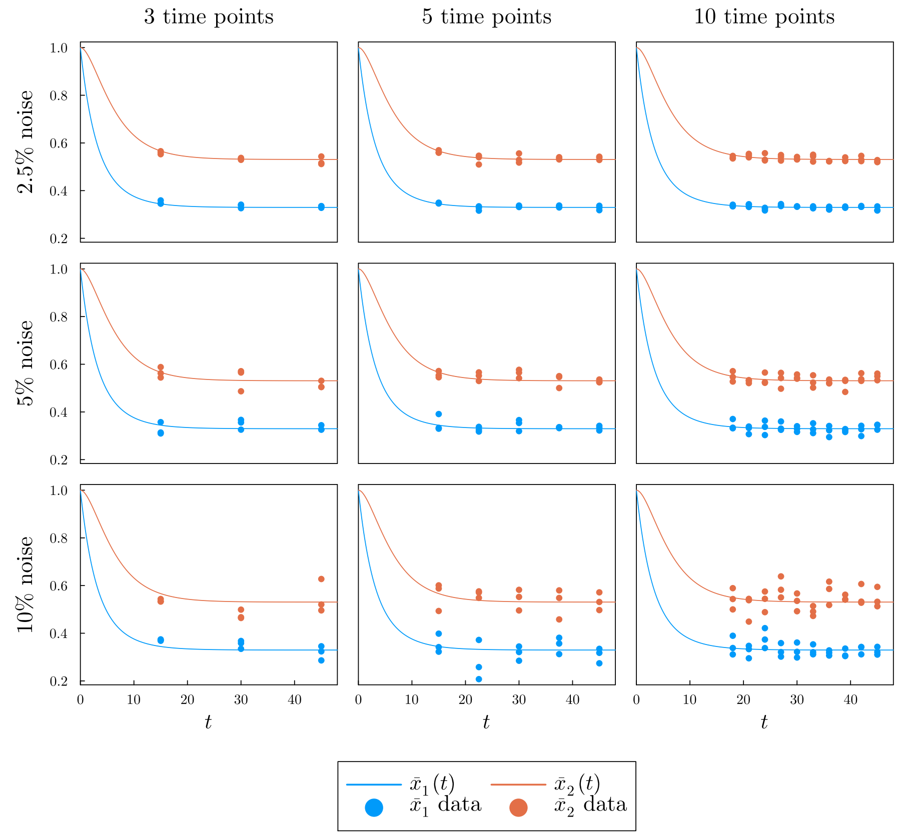

Fig. 7 Solutions and simulated noisy data for the reversible two-metabolite model with similar turnover rates when data is collected only at steady state of the system. The simulated data are taken from solutions of the system with parameter values $k_1 = 7/20$, $\alpha_1 = 1/4$, $\beta_{1,2} = 3/20$, $k_2 = 3/10$, and $\alpha_2 = 3/10$ at 3, 5, or 10 equally distributed time points. At each time point, three noisy data points are simulated by adding random normally distributed noise to the true solution with standard deviation of 2.5, 5, or 10% of the true value

In fig. 7, we see the experimental measurements are only taken while $\bar{x}_1$ and $\bar{x}_2$ approach their steady-state values. Since the experimental data captures limited information about the system we expect to get poor estimates for the turnover rates. Unfortunately, the information about the steady state is still not enough information to fully estimate the proportional parameters α_1, $\beta_{1,2}$, and α_2.

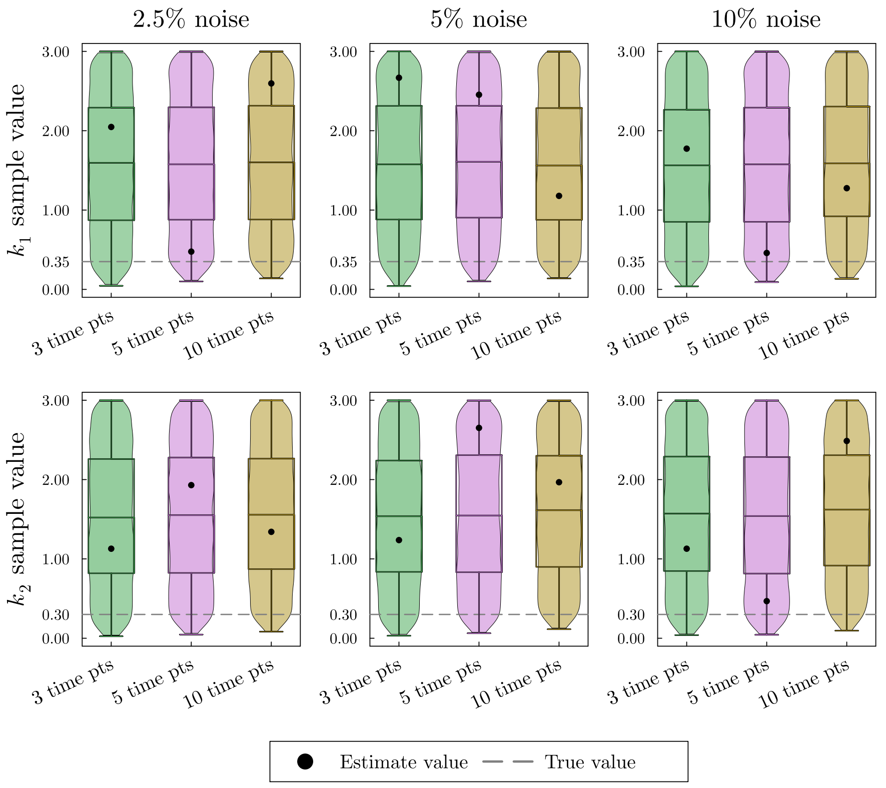

Fig. 8 Violin and box plots for posterior distributions of k_1 and k_2 for the reversible two metabolite model with similar turnover rates when data is collected only at steady state fo the system. The simulated data was taken from solution of the system with parameters $k_1 = 7/20$, $\alpha_1 = 1/4$, $\beta_{1,2} = 3/20$, $k_2 = 3/10$, $\alpha_2 = 3/10$ at 3, 5, or 10 equally spaced time points. At each time point, three noisy data points are simulated by adding random normally distributed noise to the true solution with a standard deviation of 2.5, 5, or 10% of the true value. Since k_1 and k_2 are turnover rates, we use the naive prior distributions $k_1 \sim U(0,3)$ and $k_2 \sim U(0,3)$. The mode of the posterior samples is shown as a dot and is taken as the estimated value. Outliers are excluded in the box plots

In fig. 8, we clearly see that without time points during the decay of unlabeled metabolites the Bayesian method cannot accurately estimate the turnover rates. As expected, both k_1 and k_2 have high uncertainty and in some cases very inaccurate estimates.

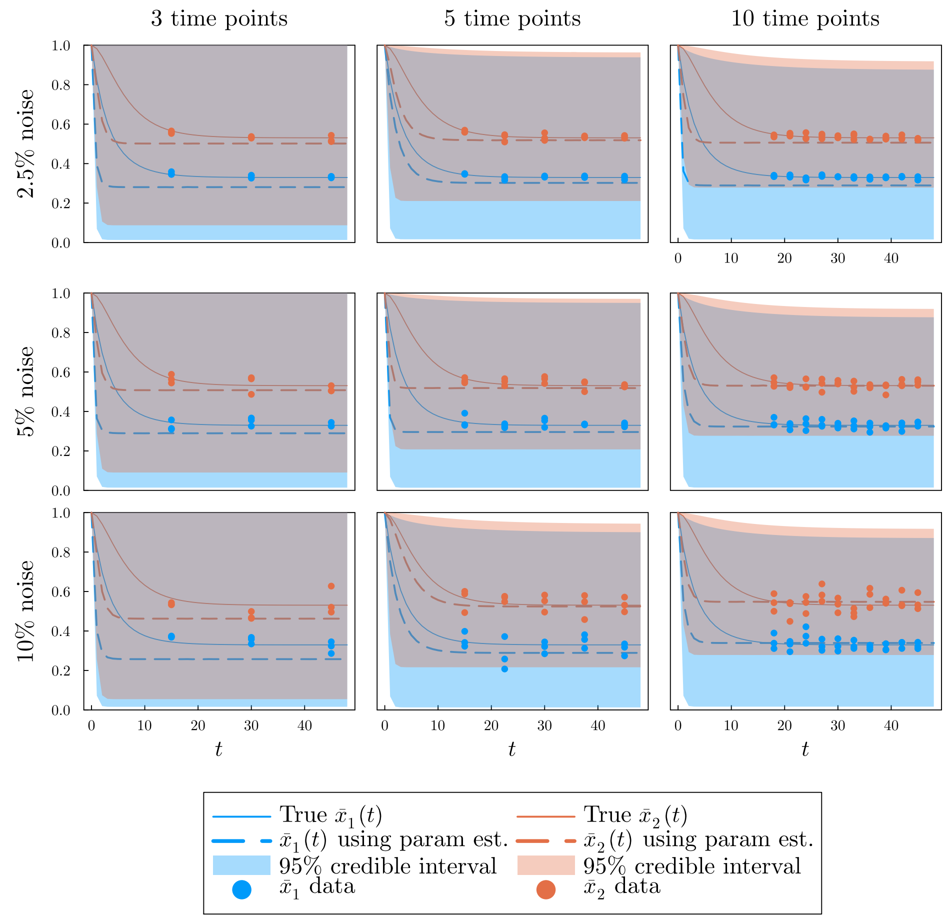

Fig. 9 Comparing solutions using the values produced from Bayesian parameter estimation to the experimental data and true solutions for the reversible two-metabolite model with similar turnover rates when data is collected only at the system's steady state. The simulated data are taken from solutions of the system with parameter $k_1 = 7/20$, $\alpha_1 = 1/4$, $\beta_{1,2} = 7/20$, $k_2 = 3/10$, $\alpha_2 = 3/10$ at 3, 5, or 10 equally distributed time points. At each time point, three noisy data points are simulated by adding random normally distributed noise to the true solution with standard deviation of 2.5, 5, or 10% of the true value. The 95% credible interval was created by plotting the region between the solution with the 2.5% and 97.5% quartiles of the posterior distributions of the parameters

In fig. 9, it appears the inaccuracy and high uncertainty in the estimate for k_1, k_2, α_1, and $\beta_{1,2}$ propagate to the solutions using the estimated parameter values. As expected, the 95% credible interval for both $\bar{x}_1$ and $\bar{x}_2$ show a large range for possible estimates.

1.2.2 Decay and Steady-State Data

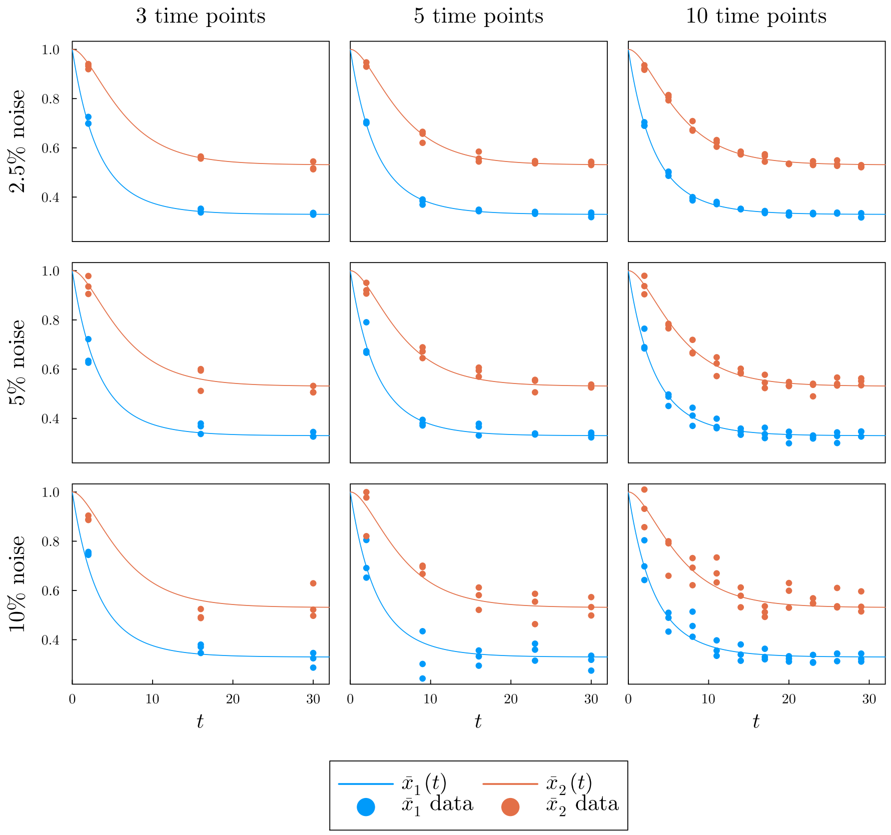

Fig. 10 Solutions and simulated noisy data for the reversible two-metabolite model with similar turnover rates. The simulated data are taken from solutions of the system with parameters $k_1 = 7/20$, $\alpha_1 = 3/10$, $\beta_{2,1} = 3/20$ $k_2 = 3/10$, $\alpha_2 = 1/4$ at 3, 5 or 10 equally distributed time points. At each time point, three noisy data points are simulated by adding random normally distributed noise to the true solutions with a standard deviation of 2.5, 5, or 10% of the true value

In fig. 10, we see that the experimental measurements are taken while both $\bar{x}_1$ and $\bar{x}_2$ are still decaying and as both approach their steady-state values. Since the experimental data captures information about the decay rates and steady-state values, we expect to get accurate estimates of all parameter values with low uncertainty given sufficient time points and little noise.

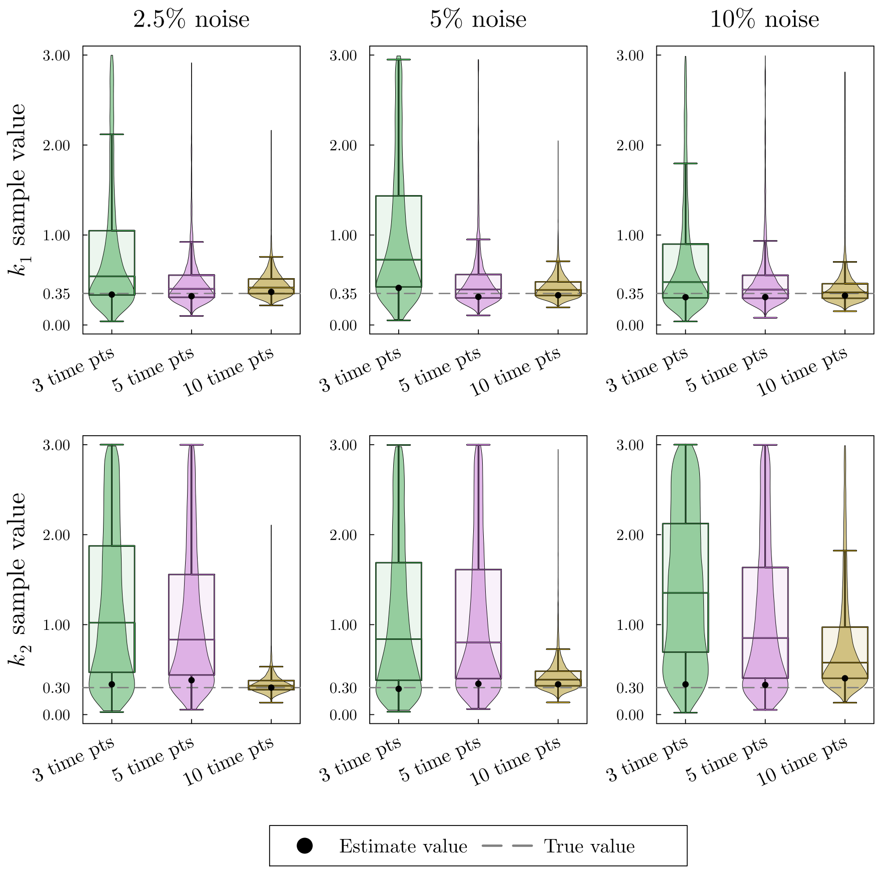

Fig. 11 Violin and box plots for posterior distributions of k_1 and k_2 for the reversible two-metabolite model with similar turnover rates. The simulated data are taken from solutions of the system with parameters $k_1 = 7/20$, $\alpha_1 = 3/10$, $\beta_{2,1} = 3/20$ $k_2 = 3/10$, $\alpha_2 = 1/4$ at 3, 5 or 10 equally distributed time points. At each time point, three noisy data points are simulated by adding random normally distributed noise to the true solutions with a standard deviation of 2.5, 5, or 10% of the true value. Since k_1, and k_2 are turnover rates, we use the naive prior distributions $k_1 \sim U(0, 3)$, $k_2 \sim U(0, 3)$. The mode of the posterior samples is shown as a dot and is taken as the estimated value. Outliers are excluded in the box plots

From fig. 11, we see that the Bayesian estimation method condensed the sample values near the true parameter values for both parameters. Regardless of the noise level, the parameter estimation increases in accuracy with the inclusion of more time points. The estimated value of the posterior distributions approaches the true parameter value as the number of time points increases. With 10 time points and 2.5% noise, we receive the best estimates with the least uncertainty for both k_1 and k_2.

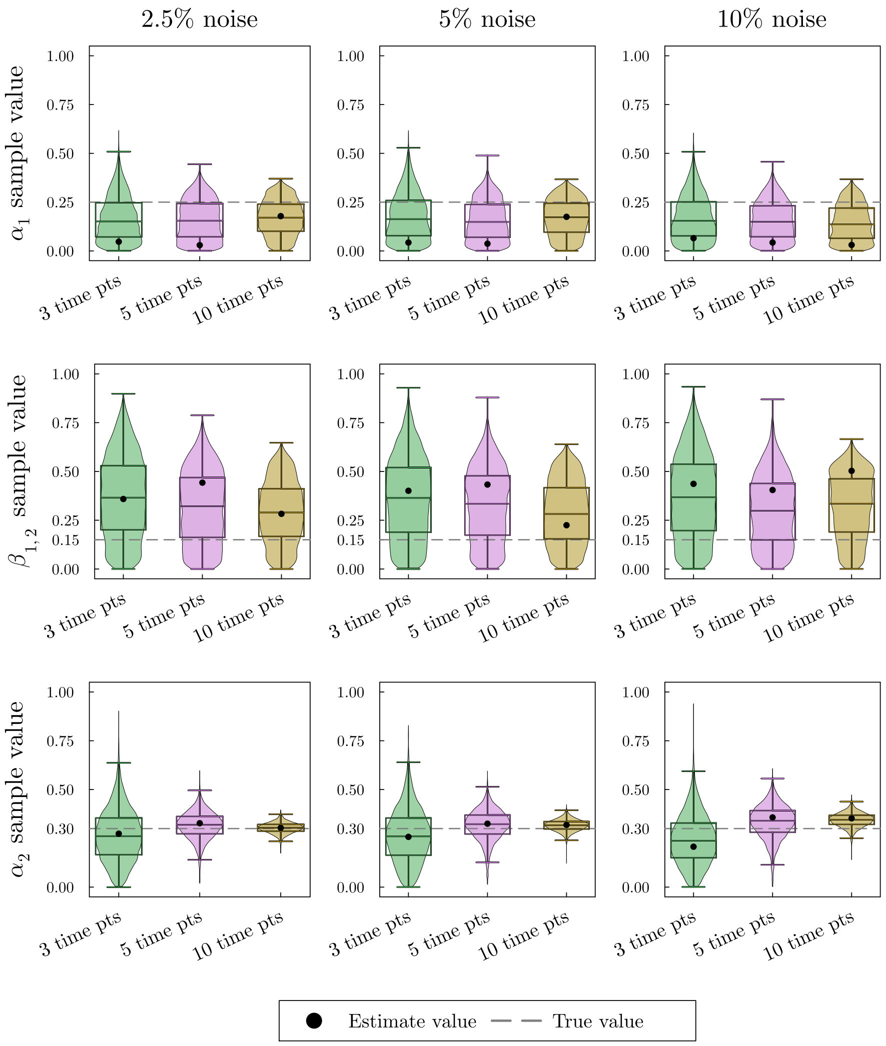

Fig. 12 Violin and box plots for posterior distributions of α_1, $\beta_{1,2}$, and α_2 for the reversible two-metabolite model with similar turnover rates. The simulated data are taken from solutions of the system with parameters $k_1 = 7/20$, $\alpha_1 = 1/4$, $\beta_{1,2} = 3/20$ $k_2 = 3/10$, $\alpha_2 = 3/10$ at 3, 5 or 10 equally distributed time points. At each time point, three noisy data points are simulated by adding random normally distributed noise to the true solutions with a standard deviation of 2.5, 5, or 10% of the true value. Since α_1, $\beta_{1,2}$, and α_2 are proportions, we use the natural naive prior distributions $\alpha_1 \sim U(0,1)$, $\beta_{1,2} \sim U(0,1)$, $\alpha_2 \sim U(0,1)$. The mode of the posterior samples is shown as a dot and is taken as the estimated value. Outliers are excluded in the box plots

In the violin plots in fig. 12, we see that we only get moderately accurate estimates for α_2. As before, as we increase the number of time points, the uncertainty for α_2 estimates decreases and the results are relatively insensitive to the magnitude of the noise. The estimates for α_1 and $\beta_{1,2}$ on the other hand are completely unsatisfactory.

The estimates of α_1 are much lower than the true value, while the estimates for $\beta_{1,2}$ are much higher than the true value. The sample distributions for both α_1 and $\beta_{1,2}$ show that there is a high uncertainty in the estimates. Since the two steady-state values depend on the values of α_1, $\beta_{1,2}$, and α_2, several combinations of parameter values give the same steady-state value. While the method may be able to correctly determine combinations of these parameters that give the correct steady-state values for both $\bar{x}_1$ and $\bar{x}_2$, it cannot discern the correct parameter values.

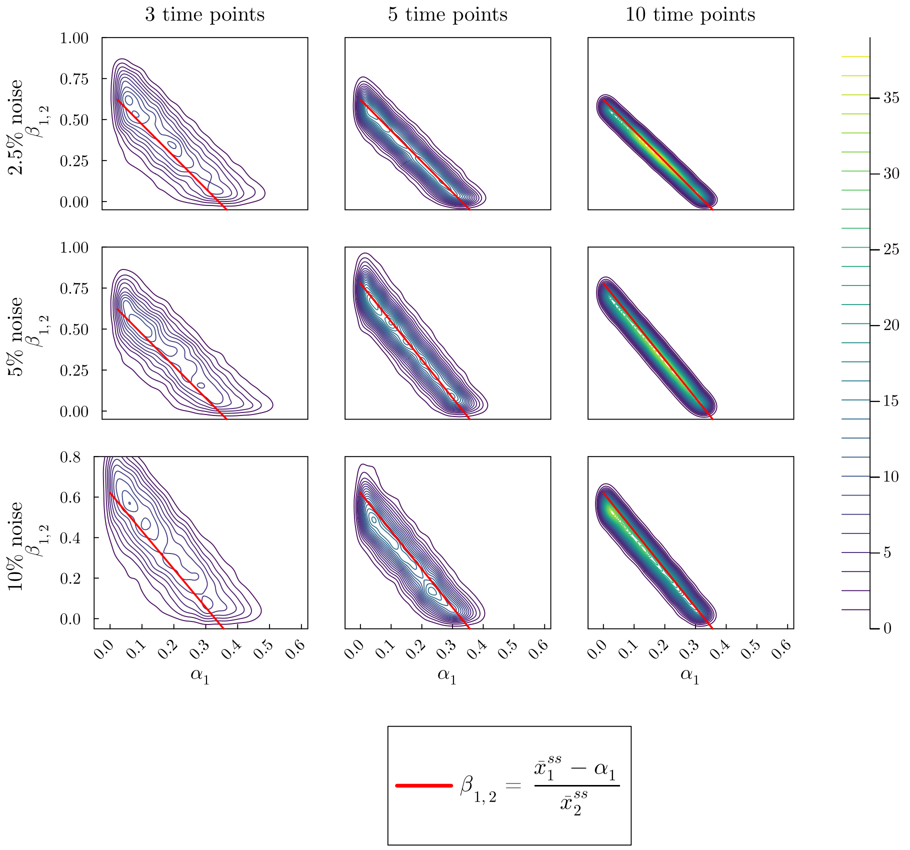

Fig. 13 Contour plot for the bivariate distribution of α_1 and $\beta_{1,2}$ for the reversible two-metabolite model with similar turnover rates. The simulated data are taken from solutions of the system with parameters $k_1 = 7/20$, $\alpha_1 = 1/4$, $\beta_{1,2} = 3/20$ $k_2 = 3/10$, $\alpha_2 = 3/10$ at 3, 5, or 10 equally distributed time points. At each time point, three noisy data points are simulated by adding random normally distributed noise to the true solutions with a standard deviation of 2.5, 5, or 10% of the true value. Since α_1 and $\beta_{1,2}$ are proportions, we use the natural naive prior distributions $\alpha_1 \sim U(0,1)$ and $\beta_{1,2} \sim U(0,1)$. The red line indicates the relationship between α_1 and $\beta_{1,2}$ with the steady-state values for $\bar{x}_1$ and $\bar{x}_2$

In the main paper, we showed that given steady-state information there exists a linear relationship between α_1 and $\beta_{1,2}$, namely $\beta_{1,2} = \frac{\bar{x}_1^{ss} - \alpha_1}{\bar{x}_2^{ss}}$ This is clearly shown

by the contour plot of the bivariate distribution of α_1 and $\beta_{1,2}$ in fig. 13. The resulting parameter estimations are condensed near the red line describing this relationship and improve with the inclusion of more time points. The individual posterior distributions of α_1 and $\beta_{1,2}$ are inaccurate and highly uncertain, but the combination of both distribution provide a better estimate of the system's steady-state.

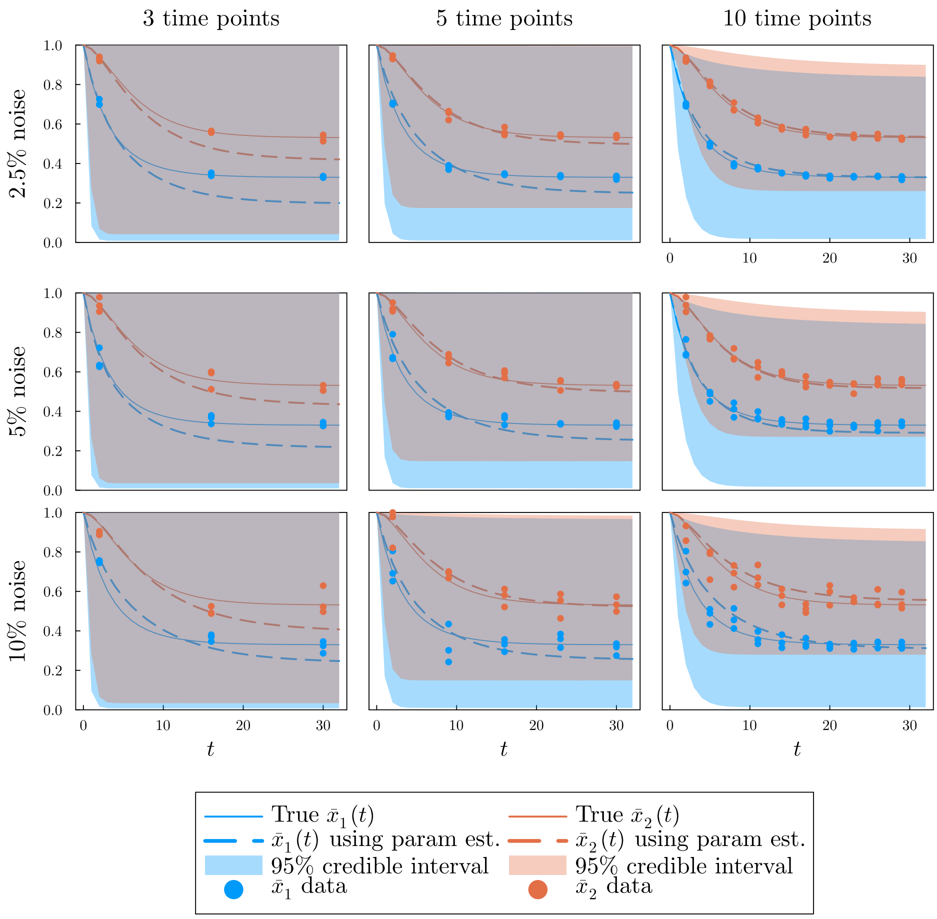

Fig. 14 Comparing solutions using the values produced from Bayesian parameter estimation to the experimental data and true solutions for the reversible two-metabolite model with similar turnover rates. The simulated data are taken from solutions of the system with parameters $k_1 = 7/20$, $\alpha_1 = 1/4$, $\beta_{1,2} = 3/20$ $k_2 = 3/10$, $\alpha_2 = 3/10$ at 3, 5 or 10 equally distributed time points. At each time point, three noisy data points are simulated by adding random normally distributed noise to the true solutions with a standard deviation of 2.5, 5, or 10% of the true value. The 95% credible interval was created by plotting the region between the solution with the 2.5% and the 97.5% quartiles of the posterior distributions of the parameters

In fig. 14, we see that the solution using the parameter estimates approaches the true solution as we increase the number of time points. The 95% credible interval

does not decrease much due to the high uncertainty in the parameter values. Given many time points, it appears that the method is accurately estimating the steady-state values for $\bar{x}_1$ and $\bar{x}_2$ while inaccurately estimating the true values of α_1 and $\beta_{1,2}$.

2 Fast-Slow Analysis - Supplementary Information

2.1 Reversible Two-Metabolite Fast-Slow Example

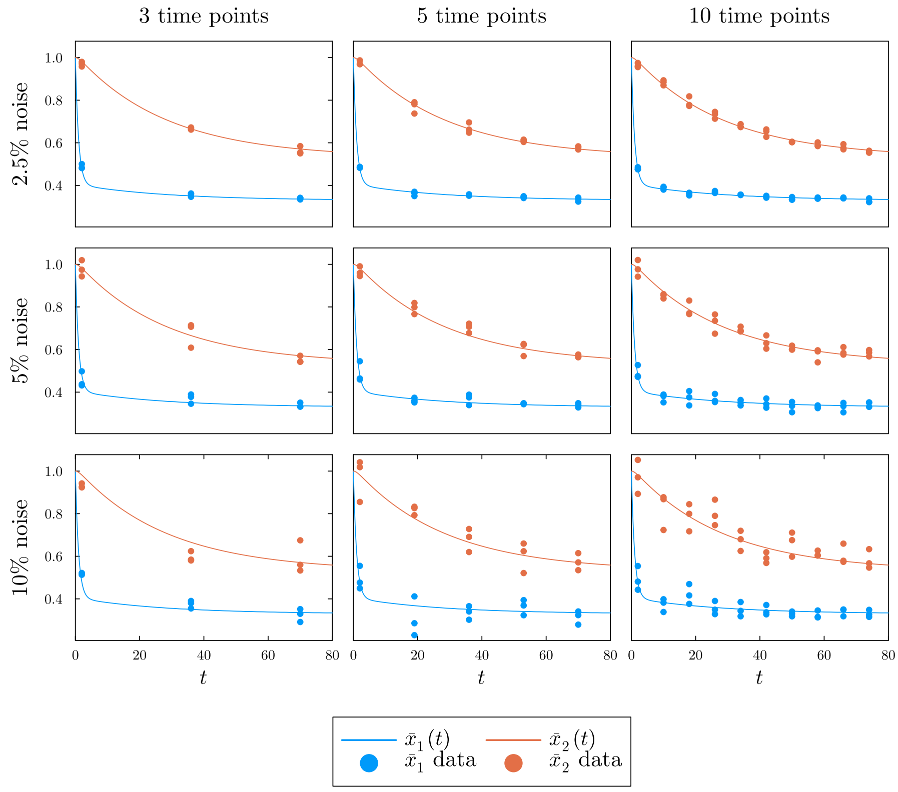

Fig. 15 Solutions and simulated noisy data for the reversible two-metabolite model with similar turnover rates. The simulated data are taken from solutions of the system with parameters $k_1 = 1$, $\alpha_1 = 1/4$, $\beta_{1,2} = 3/20$, $k_2 = 1/25$, $\alpha_2 = 3/10$ at 3, 5 or 10 equally distributed time points. At each time point, three noisy data points are simulated by adding random normally distributed noise to the true solutions with a standard deviation of 2.5, 5, or 10% of the true value

In fig. 15, we see that most of the experimental measurements are taken while $\bar{x}_!$ is near its steady-state value and $\bar{x}_2$ is still decaying. Only one of the time points is taken while $\bar{x}_1$ rapidly decays immediately after the isotope switch. Also, the time frame of the measurements does not extend far enough to truly capture $\bar{x}_2$ near its steady-state value.

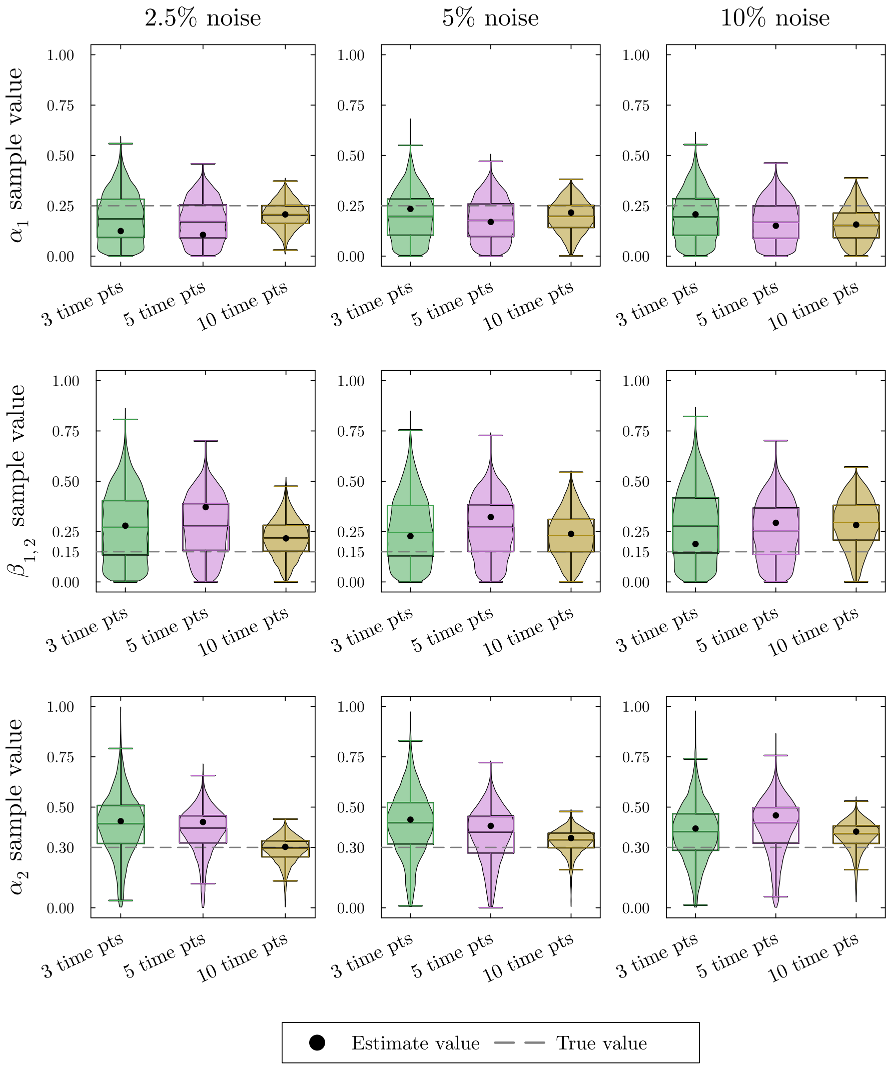

Fig. 16 Violin and box plots for posterior distributions of α_1, $beta_{1,2}$, and α_2 for the reversible two-metabolite fast-slow system. The simulated data are taken from solutions of the system with parameters $k_1 = 1$, $\alpha_1 = 1/4$, $\beta_{1,2} = 3/20$, $k_2 = 1/25$, $\alpha_2 = 3/10$ at 3, 5, or 10 equally distributed time points. At each time point, three noisy data points are simulated by adding random normally distributed noise to the true solutions with a standard deviation of 2.5, 5, or 10% of the true value. Since α_1, $\beta_{1,2}$, and α_2 are proportions, we use the natural naive prior distributions $\alpha_1 \sim U(0,1)$, $\beta_{1,2} \sim U(0,1)$, and $\alpha_2 \sim U(0,1)$. The mode of the posterior samples is shown as a dot and is taken as the estimated value. Outliers are excluded in the box plots

As expected, in fig. 19, we get poor estimates with high uncertainty for α_1 and $\beta_{1,2}$. On the bottom row of fig. 16, we see that increasing the number of time points

produces a more accurate estimate for α_2. We also note that the uncertainty in the estimation decreases with the increase in time points.

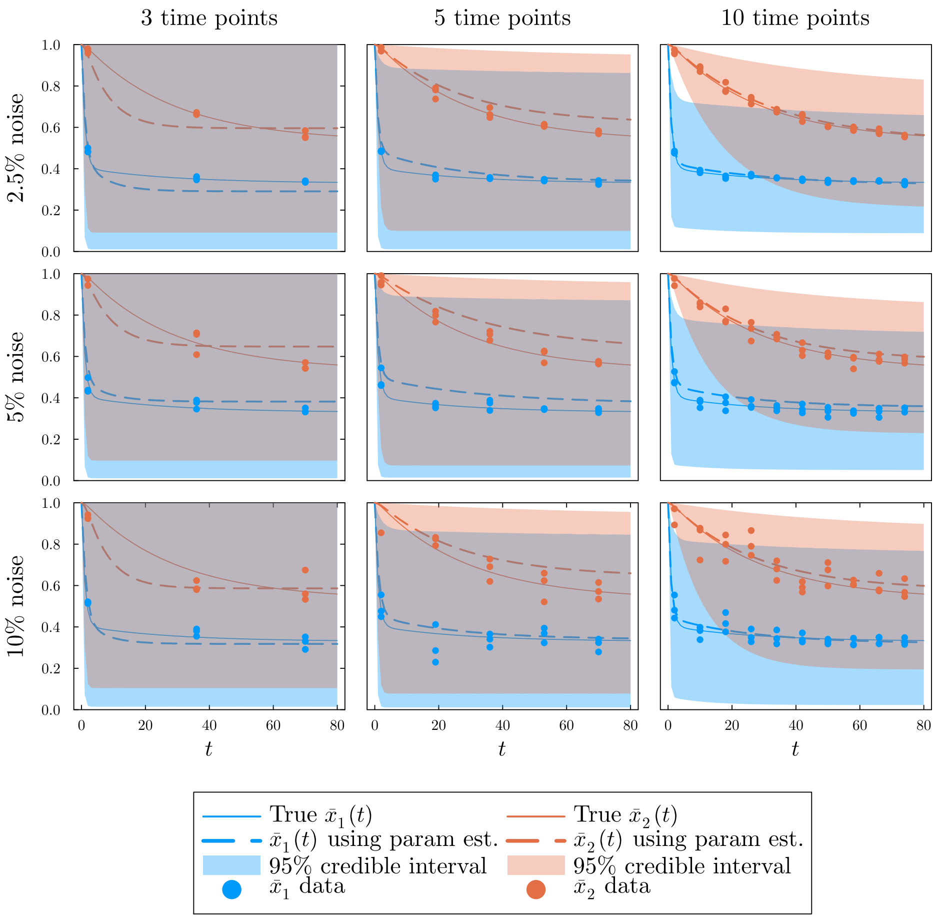

Fig. 17 Comparing solutions using the values produced from Bayesian parameter estimation to the experimental data and true solutions for the reversible two-metabolite model with similar turnover rates. The simulated data are taken from solutions of the system with parameters $k_1 = 1$, $\alpha_1 = 1/4$, $\beta_{1,2} = 3/20$, $k_2 = 1/25$, $\alpha_2 = 3/10$ at 3, 5 or 10 equally distributed time points. At each time point, three noisy data points are simulated by adding random normally distributed noise to the true solutions with a standard deviation of 2.5, 5, or 10% of the true value. The 95% credible interval was created by plotting the region between the solution with the 2.5% and the 97.5% quartiles of the posterior distributions of the parameters

In fig. 17, we see that the solution using the parameter estimates approaches the true solution as we increase the number of time points. Again, the 95% credible interval does not decrease much due to the high uncertainty in the parameter values. Given

many time points and minimal noise, it appears that the method is accurately estimating the steady-state values for $\bar{x}_1$ and $\bar{x}_2$ while inaccurately estimating the true values of α_1 and $\beta_{1,2}$.

2.2 Reversible Two-Metabolite Slow-Fast Example

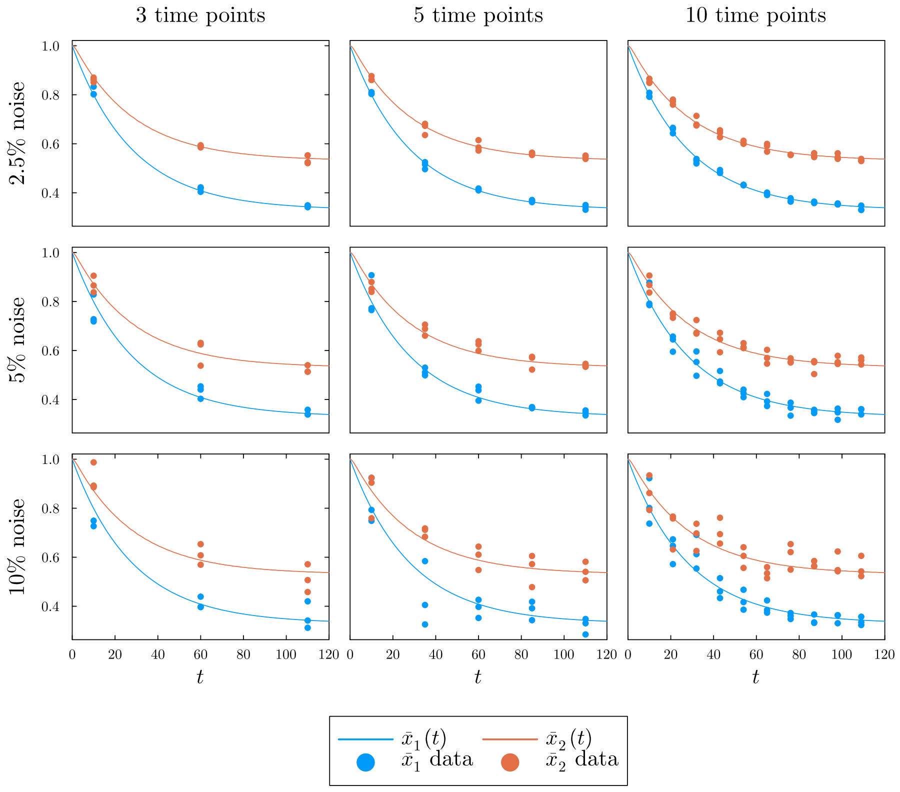

Fig. 18 Solutions and simulated noisy data for the reversible two-metabolite slow-fast system. The simulated data are taken from solutions of the system with parameters $k_1 = 1/25$, $\alpha_1 = 1/4$, $\beta_{1,2} = 3/20$, $k_2 = 1$, $\alpha_2 = 3/10$ at 3, 5, or 10 equally distributed time points. At each time point, three noisy data points are simulated by adding random normally distributed noise to the true solutions with a standard deviation of 2.5, 5, or 10% of the true value

In fig. 18, we see that the experimental measurements are taken while both $\bar{x}_1$ and $\bar{x}_2$ are still decaying and as both approach their steady-state values. In this case, we know that experimental measurements of $\bar{x}_2$ do not contain fast dynamics because the initial condition of the experiment is on the slow manifold.

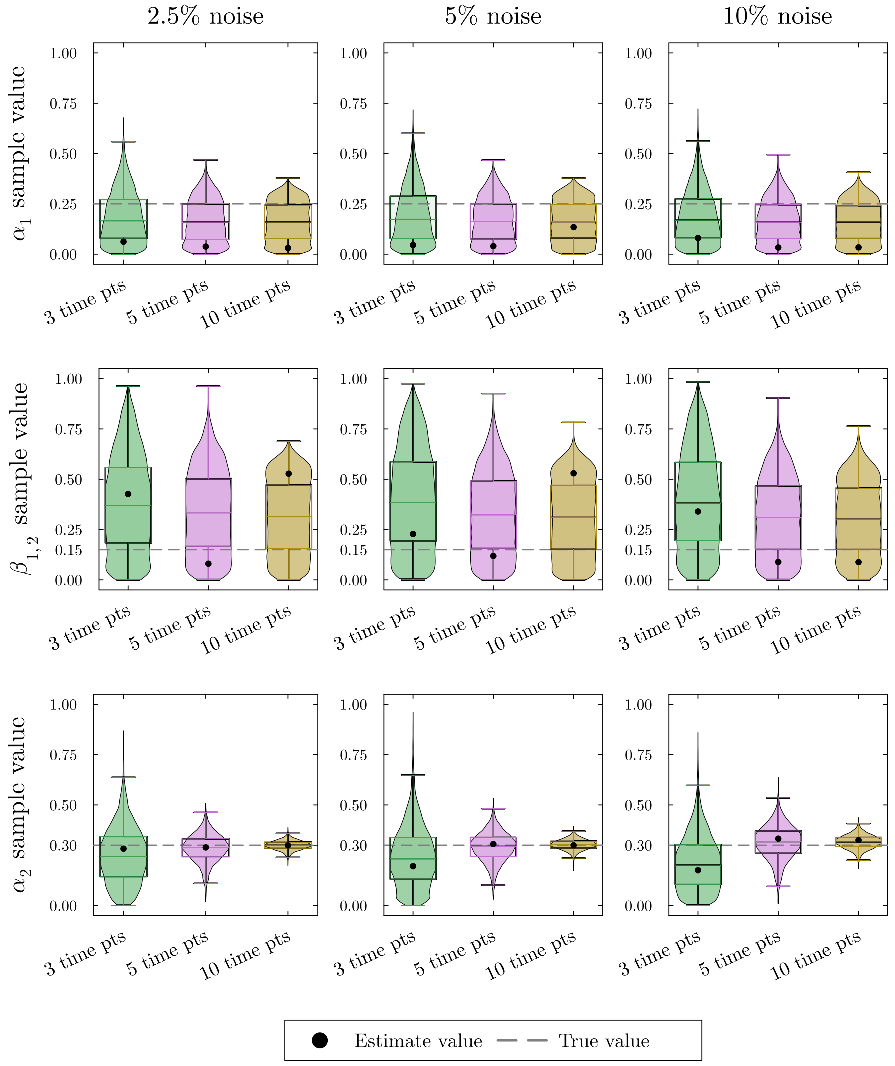

Fig. 19 Violin and box plots for posterior distributions of α_1, $beta_{1,2}$, and α_2 for the reversible two-metabolite fast-slow system. The simulated data are taken from solutions of the system with parameters $k_1 = 1$, $\alpha_1 = 1/4$, $\beta_{1,2} = 3/20$, $k_2 = 1/25$, $\alpha_2 = 3/10$ at 3, 5, or 10 equally distributed time points. At each time point, three noisy data points are simulated by adding random normally distributed noise to the true solutions with a standard deviation of 2.5, 5, or 10% of the true value. Since α_1, $\beta_{1,2}$, and α_2 are proportions, we use the natural naive prior distributions $\alpha_1 \sim U(0,1)$, $\beta_{1,2} \sim U(0,1)$, and $\alpha_2 \sim U(0,1)$. The mode of the posterior samples is shown as a dot and is taken as the estimated value. Outliers are excluded in the box plots

As expected, in fig. 19, we get poor estimates with high uncertainty for α_1 and $\beta_{1,2}$. On the bottom row of fig. 19, we see that increasing the number of time points

produces a more accurate estimate for α_2. We also note that the uncertainty in the estimation decreases with the increase in time points.

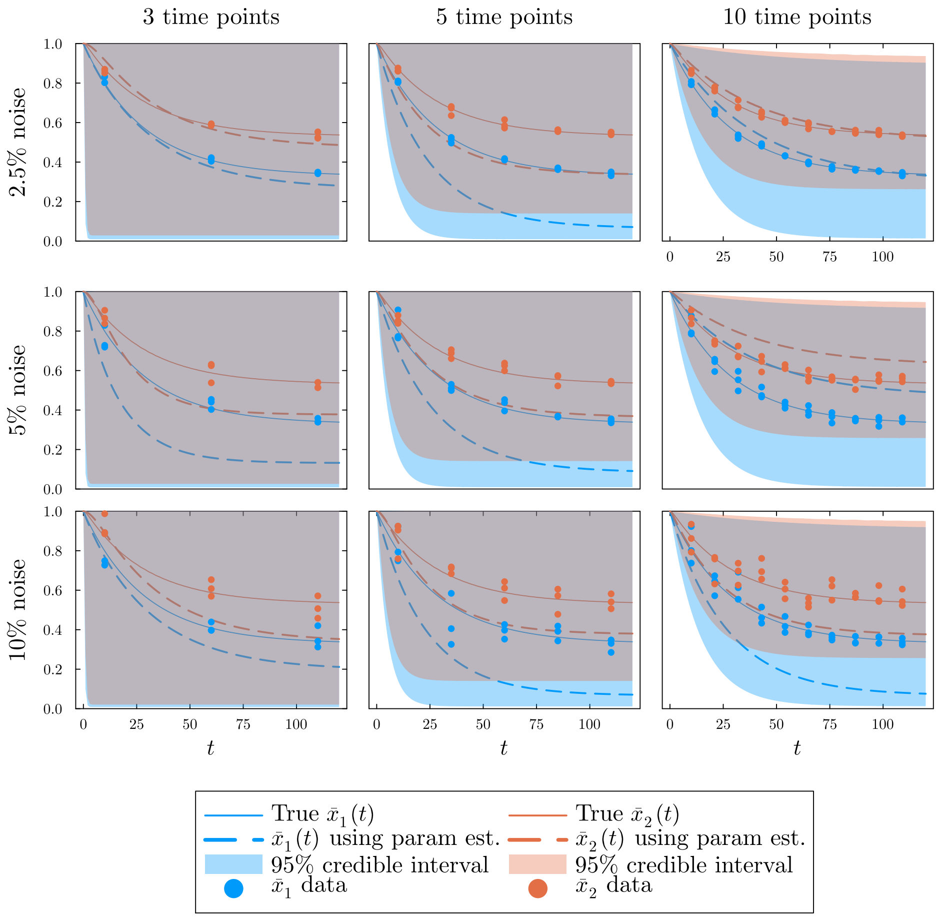

Fig. 20 Comparing solutions using the values produced from Bayesian parameter estimation to the experimental data and true solutions for the reversible two-metabolite slow-fast system. The simulated data are taken from solutions of the system with parameters $k_1 = 1/25$, $\alpha_1 = 1/4$, $\beta_{1,2} = 3/10$, $k_2 = 1$, $\alpha_2 = 3/10$ at 3, 5, or 10 equally distributed time points. At each time point, three noisy data points are simulated by adding random normally distributed noise to the true solutions with a standard deviation of 2.5, 5, or 10% of the true value. The 95% credible interval was created by plotting the region between the solution with the 2.5% and the 97.5% quartiles of the posterior distributions of the parameters

In fig. 20, it appears the inaccuracy and high uncertainty in the estimate for k_2, α_1, and $\beta_{1,2}$ show in solutions using the estimated parameter values. The 95% credible interval for both $\bar{x}_1$ and $\bar{x}_2$ show a large range for possible estimates. Overall, this case produced the worst solutions with the estimated parameter values.

2.3 Irreversible Two-Metabolite Fast-Slow Example

Consider the irreversible two-metabolite reaction network. Suppose we know the turnover rate of X_1 is much greater than the turnover rate of X_2, i.e. $k_1 \gg k_2$. Let the dimensionless variables $\tau = k_1 t$ be the fast time scale and let $\epsilon = k_2/k_1 \ll 1$ then the fast subsystem is.

$$\frac{d\bar{x}_1}{d\tau} = -\bar{x}_1 + \alpha_1$$
$$\bar{x}_2 = 1$$

Let $\eta = \epsilon\tau$ be the slow time scale to obtain the slow subsystem.

$$0 = -\bar{x}_1 + \alpha$$
$$\frac{d\bar{x}_2}{d\eta} = (1 - \alpha_2)\bar{x}_1 - \bar{x}_2 + \alpha_2$$

In this example, the slow manifold is defined by the line $\bar{x}_1 = \alpha_1$.

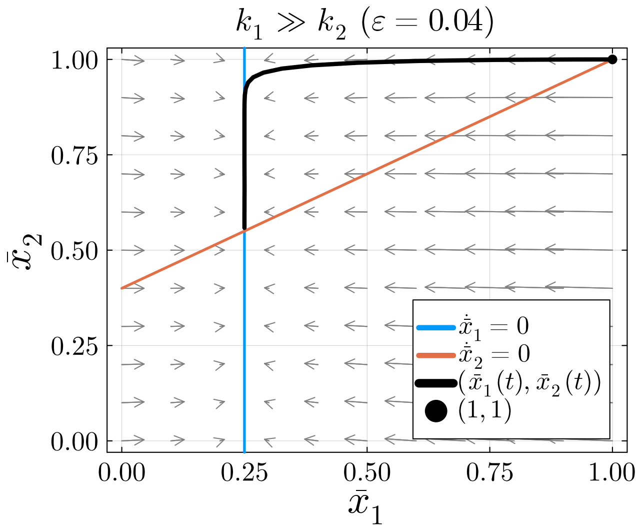

Fig. 21 The phase plane for the irreversible two-metabolite model in the case where the turnover rate of X_1 is much greater than the turnover rate of X_2. The parameter values used to create this figure are $k_1 = 1$, $\alpha_1 = 1/4$, $k_2 = 1/25$, and $\alpha_2 = 2/5$

The solution $(\bar{x}_1(t), \bar{x}_2(t))$ in fig. 21 begins at the initial condition $(1, 1)$. Next, it quickly moves from the initial condition toward the slow manifold $\bar{x}_1 = \alpha_1$. This portion of the solution shows the fast dynamics of the system. After approaching the line $\bar{x}_1 = \alpha_1$, the solution slowly moves towards the equilibrium $(\alpha_1, \alpha_1 - \alpha_1\alpha_2 + \alpha_2)$

at the intersection of $\dot{\bar{x}}_1 = 0$ and $\dot{\bar{x}}_2 = 0$. The movement along the slow manifold represents the slow dynamics of the system. In this example, data collected quickly after the isotope switch will be on the fast time scale and data collected later in the experiment will be on the slow time scale. With both early and later measurements from this biological system, KFP will provide more accurate estimations given that both fast and slow dynamics are represented.

To demonstrate parameter estimations we simulate data for this scenario as described in the appendix.

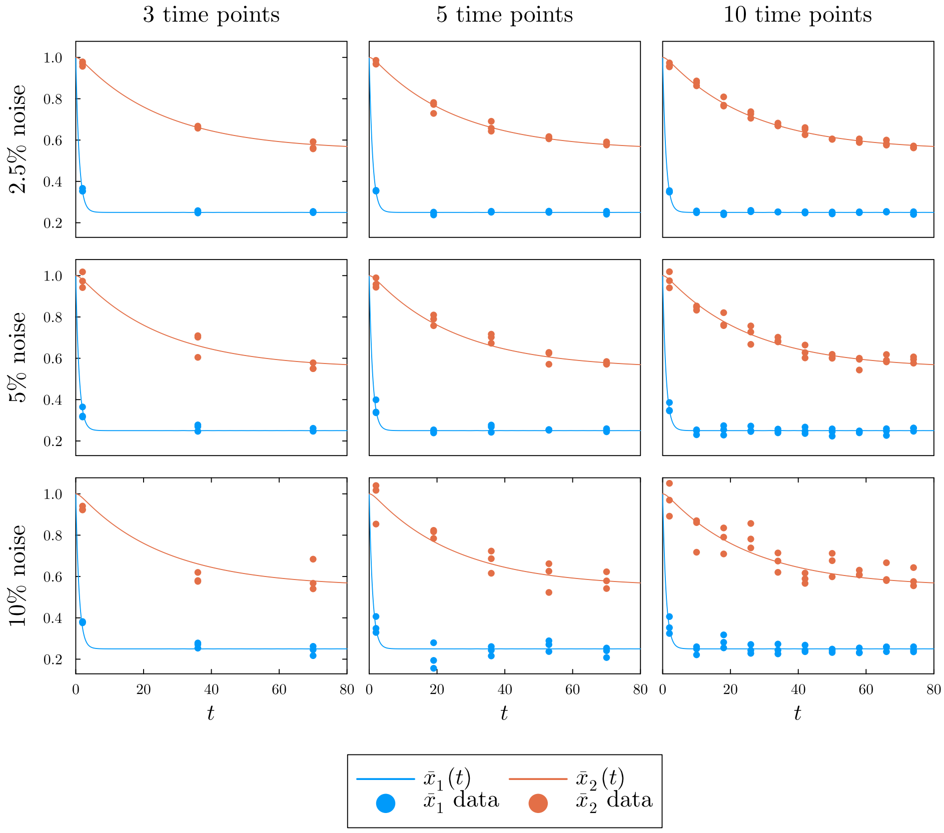

Fig. 22 Solutions and simulated noisy data for the irreversible two-metabolite fast-slow system. The simulated data are taken from solutions of the system with parameters $k_1 = 1$, $\alpha_1 = 1/4$, $k_2 = 1/25$, $\alpha_2 = 2/5$ at 3, 5, or 10 equally distributed time points. At each time point, three noisy data points are simulated by adding random normally distributed noise to the true solutions with a standard deviation of 2.5, 5, or 10% of the true value

In fig. 22, we see that most of the experimental measurements are taken while $\bar{x}_1$ is near its steady-state value, but $\bar{x}_2$ is still decaying. Only one of the time points is taken while $\bar{x}_1$ rapidly decays right after the isotope switch. Also, the time frame of

the measurements does not extend far enough to truly capture $\bar{x}_2$ near its steady-state value.

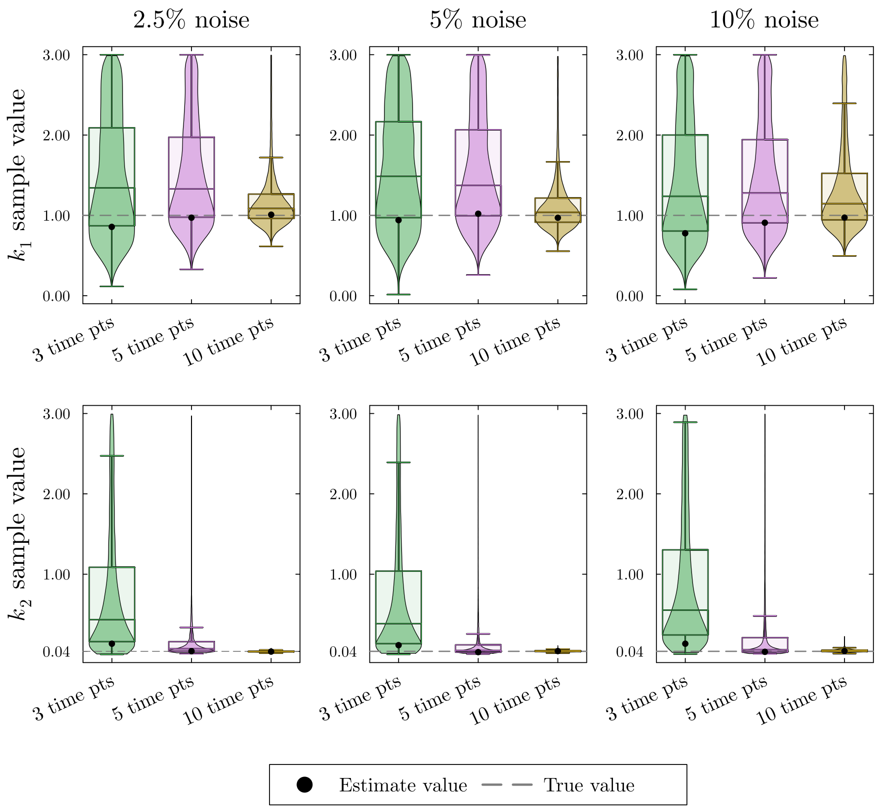

Fig. 23 Violin and box plots for posterior distributions of k_1 and k_2 for the irreversible two-metabolite fast-slow system. The simulated data are taken from solutions of the system with parameters $k_1 = 1$, $\alpha_1 = 1/4$, $k_2 = 1/25$, $\alpha_2 = 2/5$ at 3, 5, or 10 equally distributed time points. At each time point, three noisy data points are simulated by adding random normally distributed noise to the true solutions with a standard deviation of 2.5, 5, or 10% of the true value. Since k_1, and k_2 are turnover rates, we use the naive prior distributions $k_1 \sim U(0, 3)$, $k_2 \sim U(0, 3)$. The mode of the posterior samples is shown as a dot and is taken as the estimated value. Outliers are excluded in the box plots

On the top row of fig. 23, we note that the estimates for k_1 have a large uncertainty. This is because there isn't enough information on the decay rate of $\bar{x}_1$ in the experimental measurements. With only one of the time points taken during the rapid decay of $\bar{x}_1$, we cannot get a good estimate for k_1. On the bottom row of fig. 23, we see the estimate for k_2 increases in accuracy and decreases in uncertainty with the addition of time points despite the noise level. With 10 time points, the estimate for k_2 is incredibly accurate with very low uncertainty.

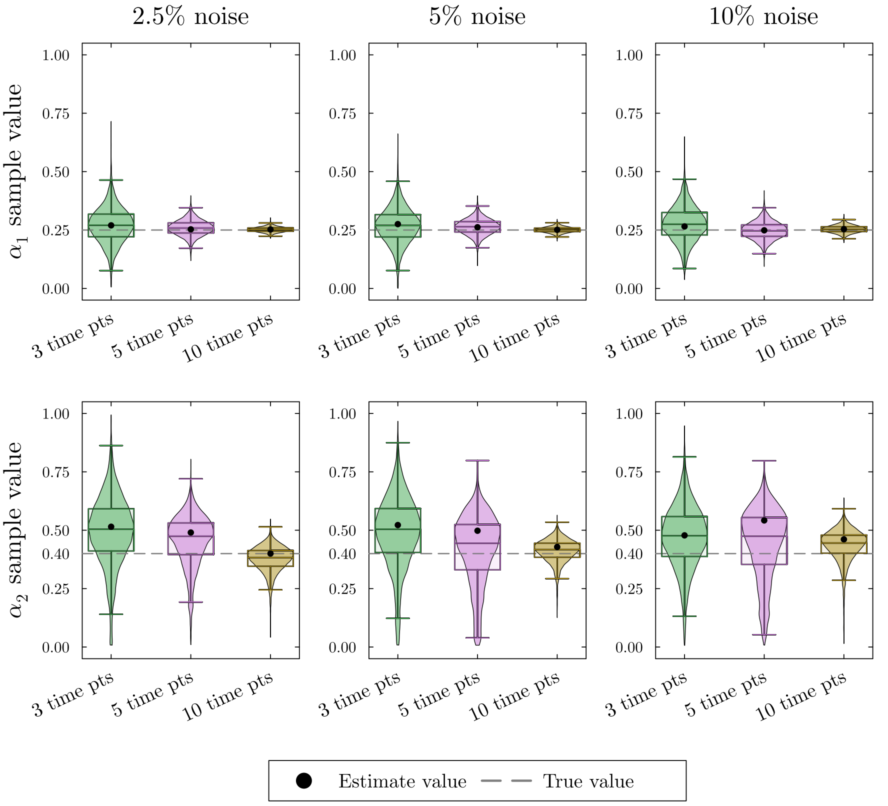

Fig. 24 Violin and box plots for posterior distributions of α_1 and α_2 for the irreversible two-metabolite fast-slow system. The simulated data are taken from solutions of the system with parameters $k_1 = 1$, $\alpha_1 = 1/4$, $k_2 = 1/25$, $\alpha_2 = 2/5$ at 3, 5, or 10 equally distributed time points. At each time point, three noisy data points are simulated by adding random normally distributed noise to the true solutions with a standard deviation of 2.5, 5, or 10% of the true value. Since α_1, and α_2 are proportions, we use the natural naive prior distributions $\alpha_1 \sim U(0, 1)$ and $\alpha_2 \sim U(0, 1)$. The mode of the posterior samples is shown as a dot and is taken as the estimated value. Outliers are excluded in the box plots

Since many of the experimental measurements are taken while $\bar{x}_1$ is near steady state, we expected to get an accurate estimate with low uncertainty for α_1 due to its direct relation to $\bar{x}_1$'s steady-state. This is clearly shown on the top row of fig. 24. Without experimental measurements of $\bar{x}_2$ near steady state, we get high uncertainty in the estimates for α_2 as shown on the bottom row of fig. 24. Without the steady-state information for $\bar{x}_2$, the Bayesian method estimates the value of α_2 to be higher than its true value.

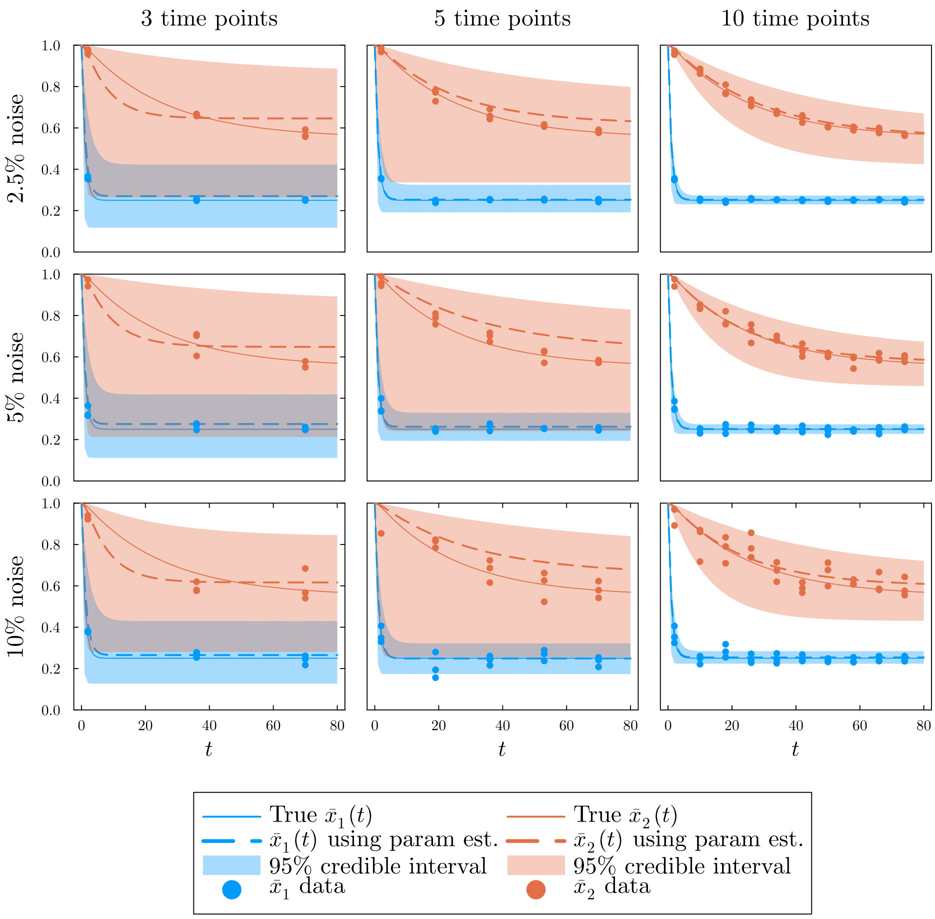

Fig. 25 Comparing solutions using the values produced from Bayesian parameter estimation to the experimental data and true solutions for the irreversible two-metabolite model fast-slow system. The simulated data are taken from solutions of the system with parameters $k_1 = 1$, $\alpha_1 = 1/4$, $k_2 = 1/25$, $\alpha_2 = 2/5$ at 3, 5, or 10 equally distributed time points. At each time point, three noisy data points are simulated by adding random normally distributed noise to the true solutions with a standard deviation of 2.5, 5, or 10% of the true value. The 95% credible interval was created by plotting the region between the solution with the 2.5% and the 97.5% quartiles of the posterior distributions of the parameters

In fig. 25, we see that the solution using the parameter estimates approaches the true solution as we increase the number of time points. In this case, the 95% credible interval for $\bar{x}_1$ does not reflect the inaccuracy and uncertainty in the estimate of k_1. The solution using the estimated parameter values looks fairly accurate. The inaccuracy and uncertainty is more obvious in the 95% credible interval for $\bar{x}_2$.

2.4 Irreversible Two-Metabolite Slow-Fast Example

Now, we will assume the opposite situation. Suppose we know the turnover rate of X_2 is much greater than the turnover rate of X_1, i.e. $k_2 \gg k_1$. Let the dimensionless variables $\tau = k_2 t$ be the fast time scale and let $\epsilon = k_1/k_2 \ll 1$. We derive the fast subsystem.

$$\begin{aligned} \bar{x}_1 &= 1 \\ \frac{d\bar{x}_2}{d\tau} &= 1 - \bar{x}_2 \end{aligned}$$

Let $\eta = \epsilon\tau$ be the slow time scale to obtain the slow subsystem.

$$\begin{aligned} \frac{d\bar{x}_1}{d\eta} &= -\bar{x}_1 + \alpha \\ 0 &= (1-\alpha_2)\bar{x}_1 - \bar{x}_2 + \alpha_2 \end{aligned}$$

In this example, the slow manifold is defined by the line $\bar{x}_2 = (1-\alpha_2)\bar{x}_1 + \alpha_2$.

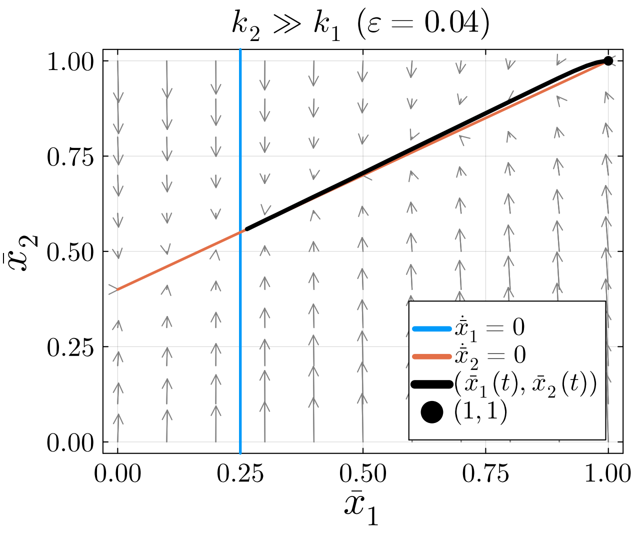

Fig. 26 The phase plane for the irreversible two-metabolite model in case where the turnover rate of X_2 is much greater than the turnover rate of X_1. The parameter values used to create this figure are $k_1 = 1/25$, $\alpha_1 = 1/4$, $k_2 = 1$, and $\alpha_2 = 2/5$

The solution $(\bar{x}_1(t), \bar{x}_2(t))$ in fig. 26 begins at the initial condition $(1, 1)$. The initial condition is located on the slow manifold $\bar{x}_2 = (1-\alpha_2)\bar{x}_1 + \alpha_2$. The solution will slowly move along the slow manifold until it approaches the equilibrium $(\alpha_1, \alpha_1 - \alpha_1\alpha_2 + \alpha_2)$ at the intersection of $\dot{\bar{x}}_1 = 0$ and $\dot{\bar{x}}_2 = 0$. Therefore, only the slow dynamics will

be visible in the experimental data. Metabolite X_1's slow turnover rate restricts the amount of label entering metabolite X_2, preventing an accurate approximation of the much faster rate through X_2. For the data to display the fast dynamics of X_2, we would need to change the initial condition of the system. Changing the initial condition would require a different experimental setup.

To demonstrate parameter estimations, we simulate data for this scenario as described in the appendix.

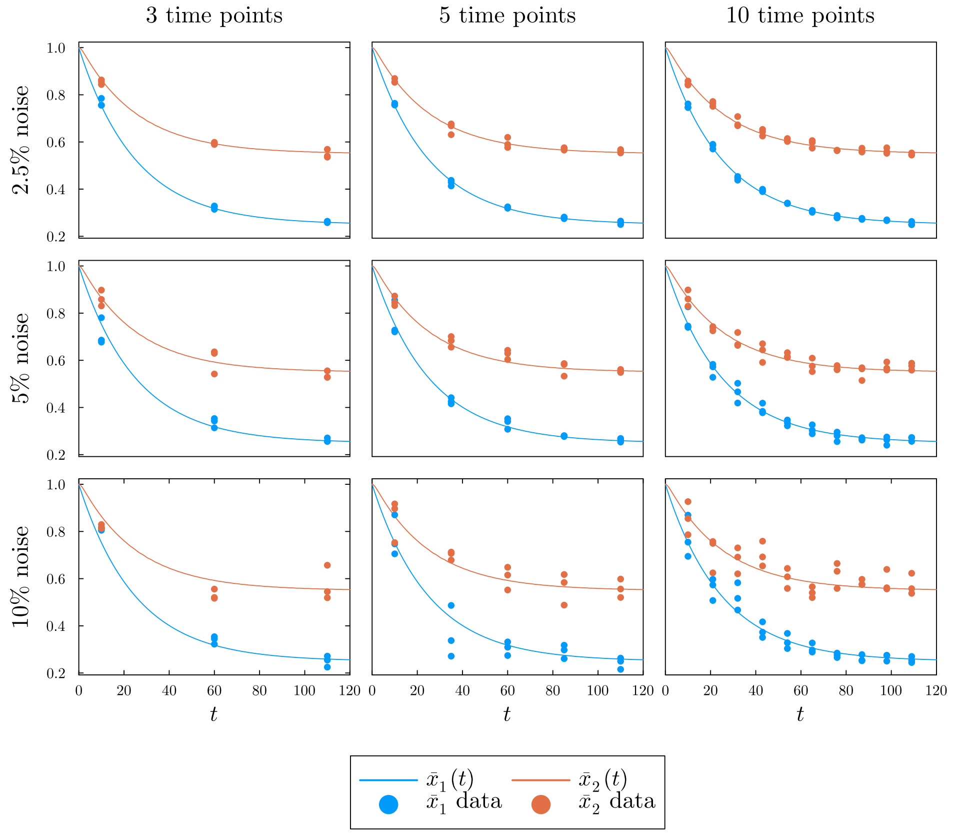

Fig. 27 Solutions and simulated noisy data for the irreversible two-metabolite slow-fast system. The simulated data are taken from solutions of the system with parameters $k_1 = 1/25$, $\alpha_1 = 1/4$, $k_2 = 1$, $\alpha_2 = 2/5$ at 3, 5, or 10 equally distributed time points. At each time point, three noisy data points are simulated by adding random normally distributed noise to the true solutions with a standard deviation of 2.5, 5, or 10% of the true value

In fig. 27, we see that the experimental measurements are taken while both $\bar{x}_1$ and $\bar{x}_2$ are still decaying and as both approach their steady-state values. In this case, we know that experimental measurements of $\bar{x}_2$ do not contain fast dynamics because the initial condition of the experiment is on the slow manifold.

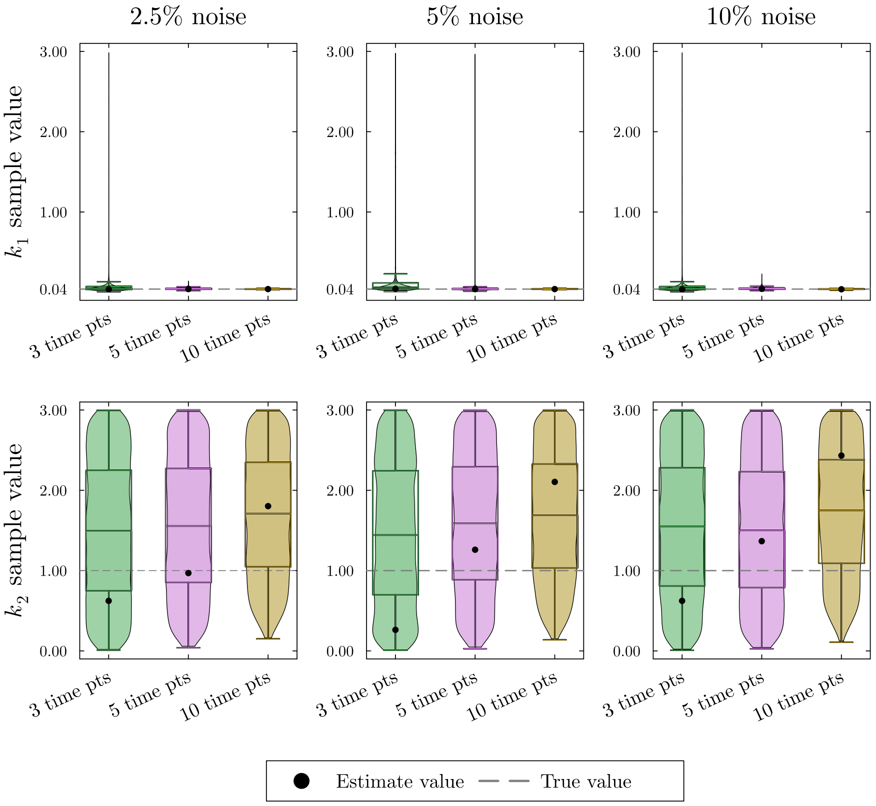

Fig. 28 Violin and box plots for posterior distributions of k_1 and k_2 for the irreversible two-metabolite slow-fast system. The simulated data are taken from solutions of the system with parameters $k_1 = 1/25$, $\alpha_1 = 1/4$, $k_2 = 1$, $\alpha_2 = 2/5$ at 3, 5, or 10 equally distributed time points. At each time point, three noisy data points are simulated by adding random normally distributed noise to the true solutions with a standard deviation of 2.5, 5, or 10% of the true value. Since k_1, and k_2 are turnover rates, we use the naive prior distributions $k_1 \sim U(0, 3)$ and $k_2 \sim U(0, 3)$. The mode of the posterior samples is shown as a dot and is taken as the estimated value. Outliers are excluded in the box plots

At the top of fig. 28, the estimates for k_1 are very accurate and have extremely low uncertainty. With many experimental measurements throughout the decay of $\bar{x}_1$, we expected to get good estimates for k_1. Contrarily, we receive poor estimates for k_2 with incredibly high uncertainty as shown at the bottom of fig. 28. The solution for $\bar{x}_2$ is not sensitive to small changes in the large k_2 value. With enough data points, Bayesian parameter estimation can rule out small values in k_2 but cannot define a good range for the true value of the parameter.

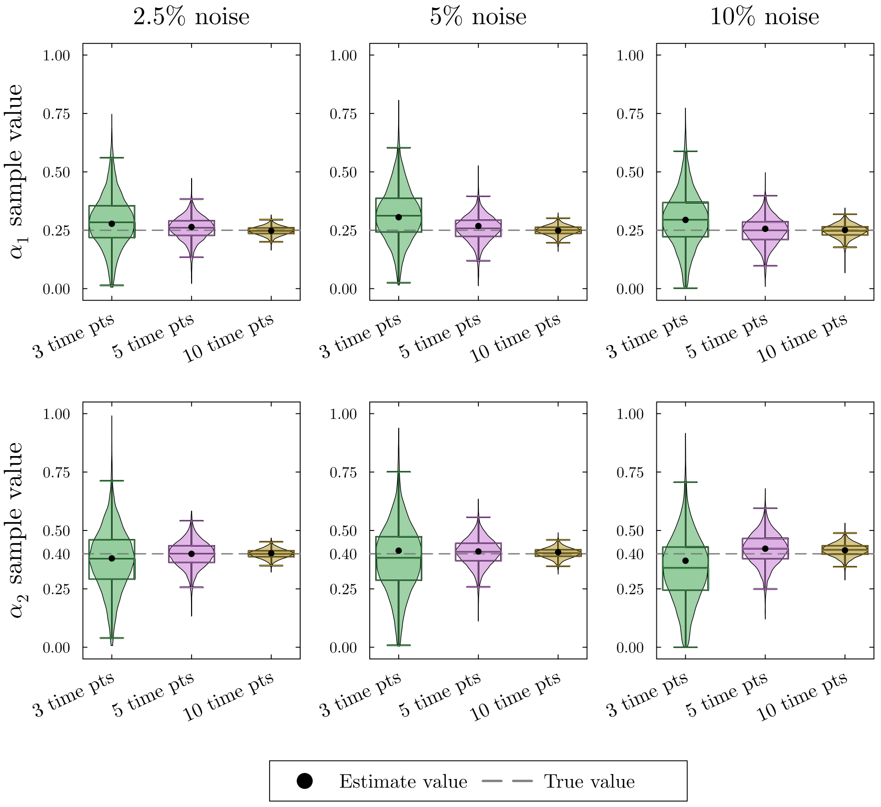

Fig. 29 Violin and box plots for posterior distributions of α_1 and α_2 for the irreversible two-metabolite slow-fast system. The simulated data are taken from solutions of the system with parameters $k_1 = 1/25$, $\alpha_1 = 1/4$, $k_2 = 1$, $\alpha_2 = 2/5$ at 3, 5, or 10 equally distributed time points. At each time point, three noisy data points are simulated by adding random normally distributed noise to the true solutions with a standard deviation of 2.5, 5, or 10% of the true value. Since α_1, and α_2 are proportions, we use the natural naive prior distributions $\alpha_1 \sim U(0, 1)$ and $\alpha_2 \sim U(0, 1)$. The mode of the posterior samples is shown as a dot and is taken as the estimated value. Outliers are excluded in the box plots

As expected, in fig. 29, we see that increasing the number of time points produces a more accurate estimate value from the samples of the posterior distributions. We also note that the uncertainty in the estimation decreases with the increase in time points. Again with 10 time points and 2.5% noise, we receive the the best estimates with the least uncertainty for both α_1 and α_2.

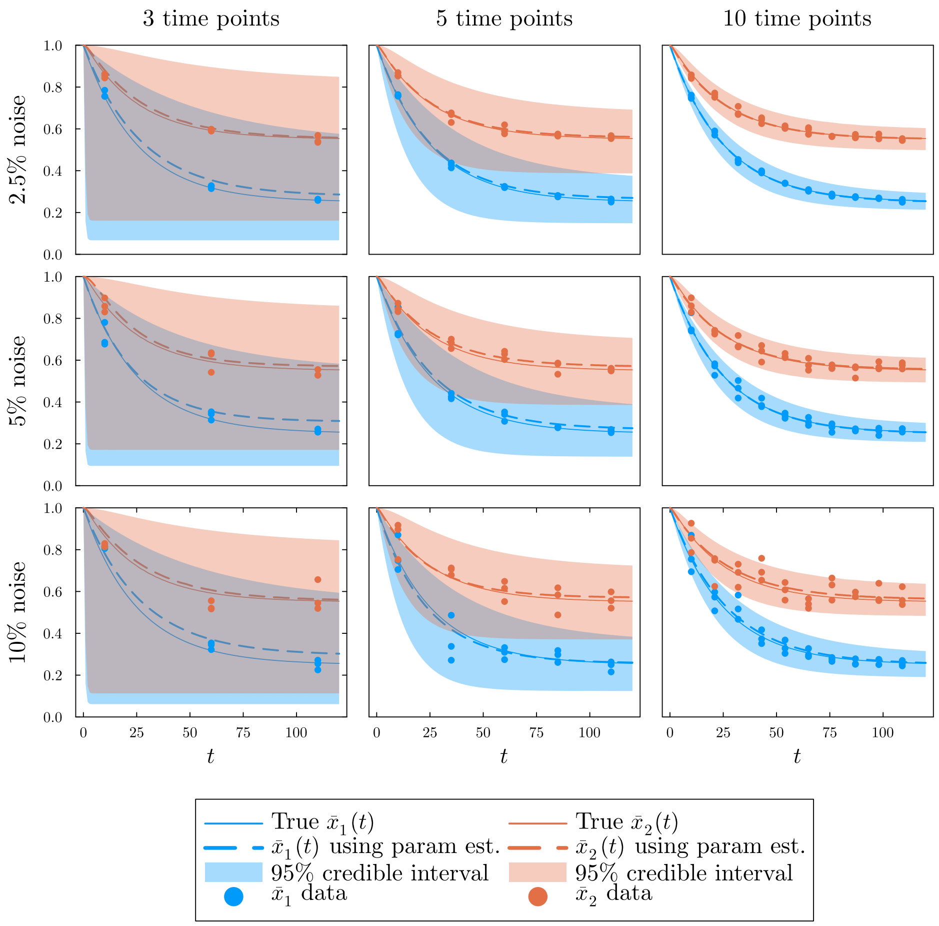

Fig. 30 Comparing solutions using the values produced from Bayesian parameter estimation to the experimental data and true solutions for the irreversible two-metabolite slow-fast system. The simulated data are taken from solutions of the system with parameters $k_1 = 1/25$, $\alpha_1 = 1/4$, $k_2 = 1$, $\alpha_2 = 2/5$ at 3, 5, or 10 equally distributed time points. At each time point, three noisy data points are simulated by adding random normally distributed noise to the true solutions with a standard deviation of 2.5, 5, or 10% of the true value. The 95% credible interval was created by plotting the region between the solution with the 2.5% and the 97.5% quartiles of the posterior distributions of the parameters

In fig. 30, it appears that the despite the inaccuracy and uncertainty in the estimate for k_2, the 95% credible interval for $\bar{x}_2$ is a small interval around the true solution. This is because the solution for $\bar{x}_2$ is not sensitive to small changes in the largest k value. Since the solution to $\bar{x}_2$ doesn't change much with the changes in the value of k_2, a wide range of k_2 values match the experimental measurements well.
